# Reconstruction of Fibroin Nanofibers (FNFs) via Electrospinning: Fabrication of Poly(vinyl alcohol)/FNFs Composite Nanofibers from Aqueous Solution

**DOI:** 10.3390/polym14010043

**Published:** 2021-12-23

**Authors:** Shohei Fujita, Huaizhong Xu, Yubing Dong, Yoko Okahisa

**Affiliations:** 1 Department of Biobased Materials Science, Kyoto Institute of Technology, Sakyoku, Kyoto 606-8585, Japan; m1661013@edu.kit.ac.jp; 2 School of Materials Science and Engineering, Zhejiang Sci-Tech University, Hangzhou 310018, China; dyb19831120@zstu.edu.cn

**Keywords:** fibroin nanofibers, electrospinning, composite nanofibers

## Abstract

Fibroin nanofibers (FNFs) achieved from physical treated silk can keep its original crystal structure, showing excellent mechanical properties, however, processing the FNFs into fibers is still a challenge. Herein, a brand-new environmentally friendly approach is proposed to manufacture FNFs-based composite nanofibers. The water-soluble polymer, poly(vinyl alcohol) PVA, was applied to increase the viscoelasticity of the spinning dope, and the content of FNFs can reach up to 20 wt%. The established phase image of spinning suggested that the concentrations ranging from 6 wt% to 8 wt% are premium to achieving relatively homogenous FNFs/PVA nanofibers. Random fibers were deposited on a fixed collector, while the fiber orientation intensity increased with the rotational speed of drum and started decreasing after 12 m/s. The mechanical properties of the composite nanofibers showed the similar tendency of variation of fiber orientation. In addition, chemical changes, crystallinity, and thermal properties of the composite nanofibers were further clarified by means of FTIR, DSC, and TG. As a result, high FNFs contained nanofibers with excellent thermal properties were created from an aqueous solution. This study is the first original work to realize the spinnability of FNFs, which provides a new insight of the FNFs.

## 1. Introduction

Silk is a well-known natural fiber that has been widely used in our daily life over thousands of years. Before obtaining the final product, silk reeling is a crucial procedure to remove the sericin component. The rest 70% part, fibroin, shows excellent mechanical properties and biocompatibility, applied in modern medicine [1]. While the fibroin filament can directly be utilized as surgical sutures [2], the regeneration and processing of the fibroin into other forms attracted lots of interests, which greatly extended its applications in the field of tissue engineering. For instance, the original fibroin can be processed to film [3], sponge [4], gel [5], or even nanofiber [6]. One of the thorny issues to regenerate the fibroin is that the fibroin cannot be dissolved in most organic solvents due to its perfect crystals of β-sheets [7]. Even if fibroin was dissolved in several certain salt solutions (e.g., Na_2_CO_3_, LiBr, CaCl_2_, etc.) [8] and ionic liquids (e.g., BMIM^+^Cl^−^, DMBIM^+^Cl^−^, EMIM^+^Cl^−^, etc.) [9], the β-sheet crystal is destroyed and shifts to random coil structure, significantly reducing its mechanical properties [10]. Moreover, the highly toxic solvent of hexafluoro-2-propanol (HFIP) was regarded as the optimal solvent to re-dissolve the regenerated fibroin [11]. Therefore, keeping the β-sheet structure of the crystal and reducing the toxicity of the solution system are the two basic scientific issues to reconstruct the fibroin for biomedical application.

Fortunately, Okahisa et al. succeeded in extracting fibroin fibrils (or fibroin nanofibers, FNFs) from silk cocoon without damaging the β-sheet crystal, and the FNFs homogeneously dispersed in water [12]. 100% FNFs-based film can be easily fabricated by vacuum filtration and drying. However, the assembly of FNFs to fibers is difficult, since the maximum high concentration to disperse the FNFs is still too low for spinning. Similar to the cellulose nanofibers (CNFs) that are directly extracted from biomass, blending with other polymers definitely improved the viscosity of the spinning dope, facilitating the spinning of CNFs-based materials [13]. This strategy should work for the FNFs. Accordingly, the water-soluble polymer, poly(vinyl alcohol) PVA, could be a nice candidate. It is a versatile degradable polymer to be utilized as tissue engineering material [14]. To date, the spinning methods include melt spinning, solution spinning, dry spinning, and gel spinning. Simultaneously, dozens of micro- and nano-technologies were developed to fabricate ultrafine fibers from various materials, such as melt blowing [15], centrifugal spinning [16], electrospinning [17], etc. Among them, electrospinning is the most appropriate method to achieve ideal nanofibers with low cost and high efficiency [18]. The porous nanofiber sheet as tissue engineering scaffold can facilitate cells’ attachment, growth, and spreading [19].

**Scheme 1:**
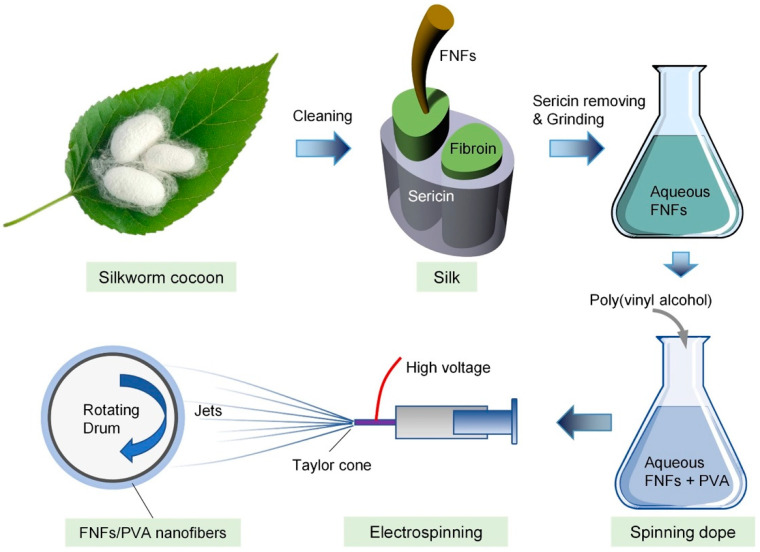
FNFs/PVA nanofibers made from electrospinning. The FNFs achieved from physical treatment can keep the β-sheet crystals of fibroin that show excellent mechanical properties. Herein, the FNFs were first fabricated into fibers with the combination of PVA.

In this study, FNFs were first reassembled into nanofibers through electrospinning. PVA was utilized as the thickener to manipulate the viscoelasticity of the mixed spinning dope. The schematic image of the process is shown in Scheme 1. The main purpose of the study is to define the spinnability of the FNFs/PVA composite nanofibers, as well as to enhance the mechanical and thermal properties of the composite fibers. This fundamental work provides a new insight into the FNFs as a new biobased material that can be reused.

## 2. Materials and Methods

### 2.1. Materials

Raw silk was degummed with a 0.9 wt% Na_2_CO_3_ aqueous solution at 95 °C for 2 h to fully remove the covered sericin. After rinsing with distilled water, purified silk fibroin was obtained. FNFs slurry was prepared after 1 wt% dispersed silk fibroin passed through a grinder running at a speed of 1500 rpm for four times (MKCA6-3, Masuko Sangyo Co., Ltd., Kawaguchi, Japan).

### 2.2. Preparation of FNFs/PVA Slurry

The FNFs slurry was concentrated in an evaporator to a certain concentration that can homogeneously disperse the FNFs. PVA powders were then dissolved in the concentrated FNFs slurry. The FNFs-to-PVA ratios (wt./wt.) were prepared at 0/10, 1/9, and 2/8, abbreviated as FP-0/10, FP-1/9, and FP-2/8. The concentration of PVA in each FNFs/PVA solution was adjusted to be 4 wt%, 6 wt%, 8 wt%, and 10 wt%.

### 2.3. Electrospinning of FNFs/PVA Solution

The FNFs/PVA solution was added into a 1-mL plastic syringe (SS-01T, TERUMO, Corp., Tokyo, Japan), connected with a 23-gauge needle. A positive voltage of 15 kV (Model-600F, Pulse Electronic Engineering Co., Ltd., Node, Japan) was loaded to the needle. The FNFs/PVA slurry was extruded out from the needle at a flow rate of 2 mL/hour by means of a micro feeder (JP-N, Sanyo Technos, Ltd., Kawaguchi, Japan). A flat stainless-steel plate was applied to collect nonwoven fibers and a rotational drum was used to collect oriented fibers. The linear velocity of the rotating drum was set at 3 m/s, 6 m/s, 9 m/s, 12 m/s, and 15 m/s. The nozzle-to-collector distance was fixed at 10 cm. During electrospinning, the room temperature was controlled at 23 °C to 24 °C and the relative humidity was controlled at 37% to 42%.

### 2.4. Characterization of Nanofiber Membrane

#### 2.4.1. Scanning Electron Microscope

The morphology of the nanofibers was investigated via a scanning electron microscope (SEM, Hitachi High-Tech Science Co., Tokyo, Japan) at a voltage of 10 kV after Au sputtering (IB-2, Eiko Engineering, Co., Ltd., Tokyo, Japan) at a current of 7 mA for 5 min.

#### 2.4.2. Mechanical Properties

The composite nanofiber membrane was peeled off from collector and rolled into a thick thread for tensile test by means of a universal material testing machine (EZSX, Shimadzu, Kyoto, Japan). The elongation rate was set at 10 mm/min. Before testing, the samples were dried in a desiccator at room temperature for over 12 h. The tensile tests were carried out at room temperature of 24 °C and relative humidity of 50%. Each measurement was repeated six times.

#### 2.4.3. Fourier Transform Infrared

The chemical change was evaluated from a Fourier transform infrared spectroscopy (FTIR, Spectrum One, PerkinElmer, Hongkong, China) with the mode of attenuated total reflection (ATR). The FTIR spectra were recorded from 4000 to 550 cm^−1^ with an average of 64 scans and a resolution of 4 cm^−1^.

#### 2.4.4. Thermal Properties

Differential scanning calorimetry (DSC) was performed on the equipment of DSC3100SA (Zetzsch, Yokohama, Japan) at a temperature rising rate of 10 °C/min from room temperature to 250 °C in a nitrogen atmosphere.

Thermogravimetric (TG) and derivative thermogravimetric (DTG) analyses (STA7200RV, Hitachi High-Tech Science Co., Japan) were carried out from 100 °C to 600 °C at a temperature increase rate of 10 °C/min after a dehydration process to remove the free water within the FNFs at 100 °C for 20 min. The process was carried out in a nitrogen atmosphere at a flow rate of 60 mL/min.

#### 2.4.5. Wide-Angle X-ray Diffraction (WAXD)

A wide-angle X-ray diffractometer (CN4037A1, Rigaku, Akishima, Japan) was used for higher-order structural analysis of the FNFs/fibroin composite nanofibers. The diffraction pattern was recorded on an imaging plate (IP). The voltage and the current of the tube were set at 40 kV and 20 mA, respectively. CuKα ray (wavelength = 0.1542 nm) filtered with Ni irradiated the samples for 30 min. An IP reader (R-AXIS DS3A, Rigaku) was applied to scan the intensity of the diffraction. The distance between the sample and the IP was set to 60.14 mm.

## 3. Results and Discussion

### 3.1. Morphology of the FNFs/PVA Composite Nanofibers

The fiber diameter of the pure FNFs applied here ranges from 150 to 200 nm [12]. While FNF itself belongs to nanofiber, the FNFs compactly stuck together after casting due to its excellent hydrophilicity, forming a transparent film. Electrospinning is capable of reconstructing the FNFs in a reasonable processing condition. Generally, the spinnability (capability of being spun) of polymeric mixture decreases with increasing the percentage of the fillers. Hence, balancing the FNFs content and the properties of the composite fibers is crucial in this study.

**Figure 1:**
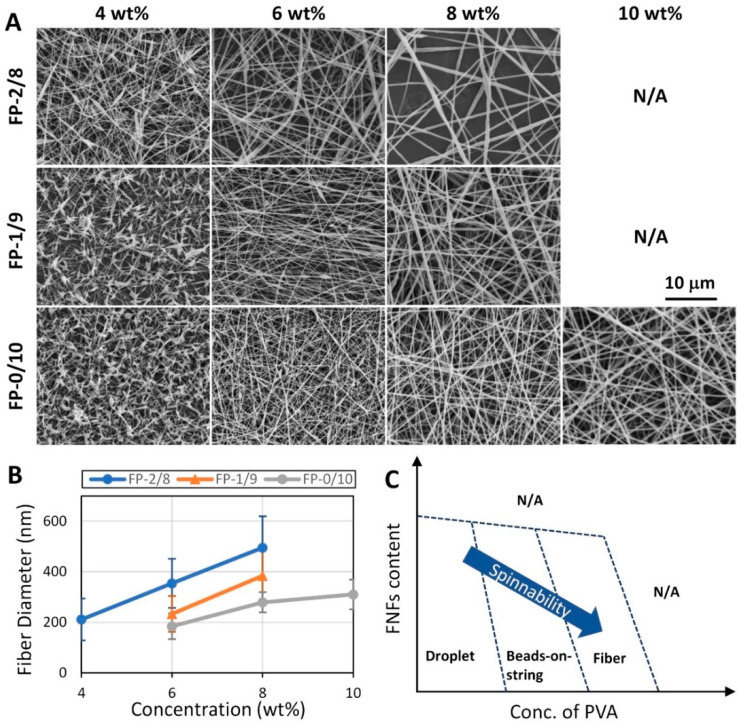
(**A**) Morphologies of FNFs/PVA nanofibers produced at concentrations of 4, 6, 8, and 10 wt%, and (**B**) the corresponding fiber diameters. (**C**) Phase diagram of the spinnability for the FNFs/PVA spinning dopes.

Figure 1A shows the SEM images of the as-spun FNFs/PVA fibers. Beads-on-string structure was formed at the concentration of 4 wt%, while it was unexpected to find that the beads content decreased with increasing the proportion of the FNFs. At low concentration, Plateau-Rayleigh instability easily results in beaded fibers that can be improved by increasing the polymeric viscosity. Unlike spherical fillers, the slender FNFs could facilitate the entanglements of the molecular chains of PVA and the FNFs. It should be noted that the spinnability drastically gets worse when the FNFs-to-PVA ratio was larger than 2/8. In addition to polymeric viscosity, the elasticity is an indispensable part for spinning, while here the FNFs without sufficient elastic properties restrain jet from being elongated to thin fibers. The beads-on-string structure shifted to fibers when the concentration of PVA was larger than 6 wt%. It is clear that the fiber diameter increased with increasing solution concentration and the proportion of the FNFs (Figure 1B). The minimum fiber diameter can be as low as 200 nm and the maximum fiber diameter was approximately 500 nm. This surprising discovery indicates that the nanoscale FNFs is possible to form the same scale composite fibers without special chemical treatment.

For the sample of FP-2/8 at 8 wt%, few fibers were detected after 10-min spinning. In this condition, the shape of Taylor-cone (the beginning of the jet) was quite unstable, that is, the Taylor-cone broke up and randomly ejected forwards or even backwards. The high content of FNFs resulted in the heterogeneous FNFs/PVA/water ternary solution at Taylor-cone, and thus the deviation of fiber diameter increased with adding of FNFs. When the concentration of PVA was adjusted to 10 wt%, large broken jets were formed instead of the composite fibers, because the viscoelasticity of the spinning dope was not fit for spinning any more. Therefore, the parameters of FP-2/8 at 6 wt% and FP-1/9 at 8 wt% are optimal to process relatively nice FNFs-based nanofibers.

To clarify the spinnable regimes, a phase diagram is established according to a large number of experiments, as shown in Figure 1C. Electrospraying occurs at low concentration, and instead of continuous jet, droplets will be formed during spinning. It is a convenient method to obtain FNFs nanoparticles. While the window for fiber formation is limited, taming the shape of Taylor-cone can achieve ideal fibers rapidly. The arrow in the figure exhibits the tendency of the spinnability, that is, relatively high concentration of PVA and low content of FNFs are beneficial for achieving fibers.

The WAXD results in Figure S1 (supporting information) reflect the crystal information of the FNFs film, PVA, and their composite nanofibers. The FNFs film shows the characterization peaks of β-sheet crystal at 20.6° (silk II) and 24.4° (silk I). Although the peak of silk II was overlapped with the main peak of PVA, a small peak can be observed at 24.4° for the samples of FP-1/9 and FP-2/8, indicating that the original crystal form will be kept after electrospinning.

**Figure 2:**
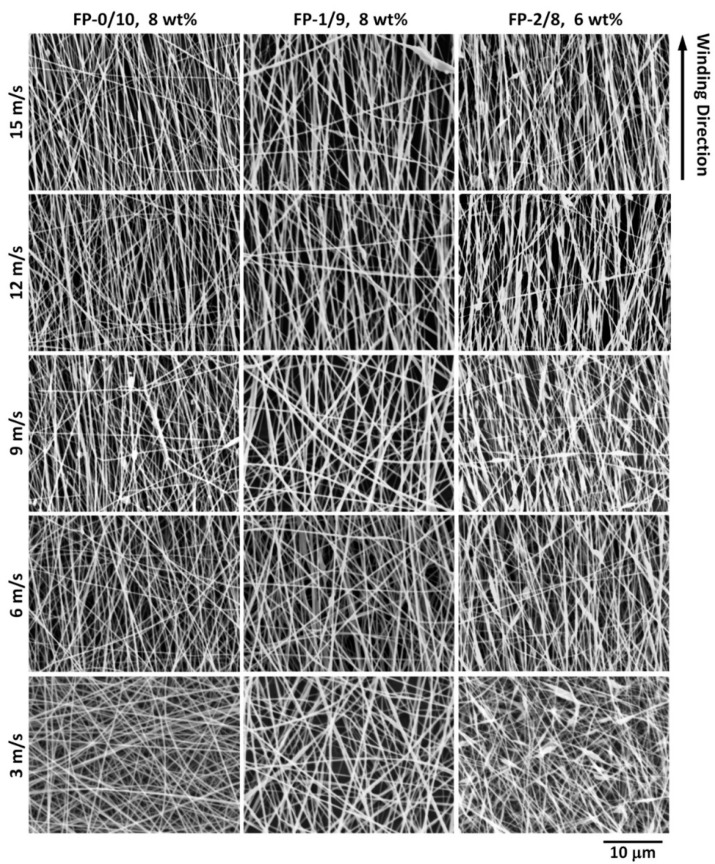
Oriented fibers produced from the spinning dopes of FP-0/10 at 8 wt%, FP-1/9 at 8 wt%, and FP-2/8 at 6 wt% were collected at rotational speeds of 3, 6, 9, 12, and 15 mm/s.

The as-spun fibers may not be stretched sufficiently for the short nozzle-to-collector distance, and thus a high-speed drum was applied to enhance the orientation of the FNFs within fibers as well as the orientation of the PVA molecular chains. When the rotational speed was larger than the jet speed, fibers will be stretched to the drawing direction. Otherwise, fibers show a certain degree to the drawing direction. The spinning dopes of FP-0/10 at 8 wt%, FP-1/9 at 8 wt%, and FP-2/8 at 6 wt% were spun at rotational speeds of 3, 6, 9, 12, and 15 m/s, and the fiber morphologies are displayed in Figure 2. Fiber diameter does not show a clear change with increasing the rotational speed, while beads-on-string fibers formed at high fibroin content (FP-2/8), which is related with the stability of the Taylor-cone during spinning.

To numerically show the orientation intensity, the images in Figure 2 were shifted to binary images (Figure S2, supporting information), followed by a Fourier transform [20]. Figure S3 (supporting information) shows the relationship between the rotational speed and the orientation intensity. At low rotational speed (3 m/s), the orientation intensities of the fibers are close to 1.2 that means a fraction of fibers starts to be aligned. For the same spinning dope, the orientation intensity does not show clear change when the rotational speed increases from 6 to 9 m/s. At 12 m/s, the fiber orientation can reach up to 1.8 and then decreased. In addition to the rotational speed, air flow also strongly affects fiber distribution. The air flow caused by the rotating drum increases with the rotational speed, which may destroy the fiber orientation. Therefore, the complex spinning conditions result in the nonlinear variation of the fiber orientation with increasing the rotational speed.

### 3.2. Tensile Properties of the FNF/PVA Nanofibers

**Figure 3:**
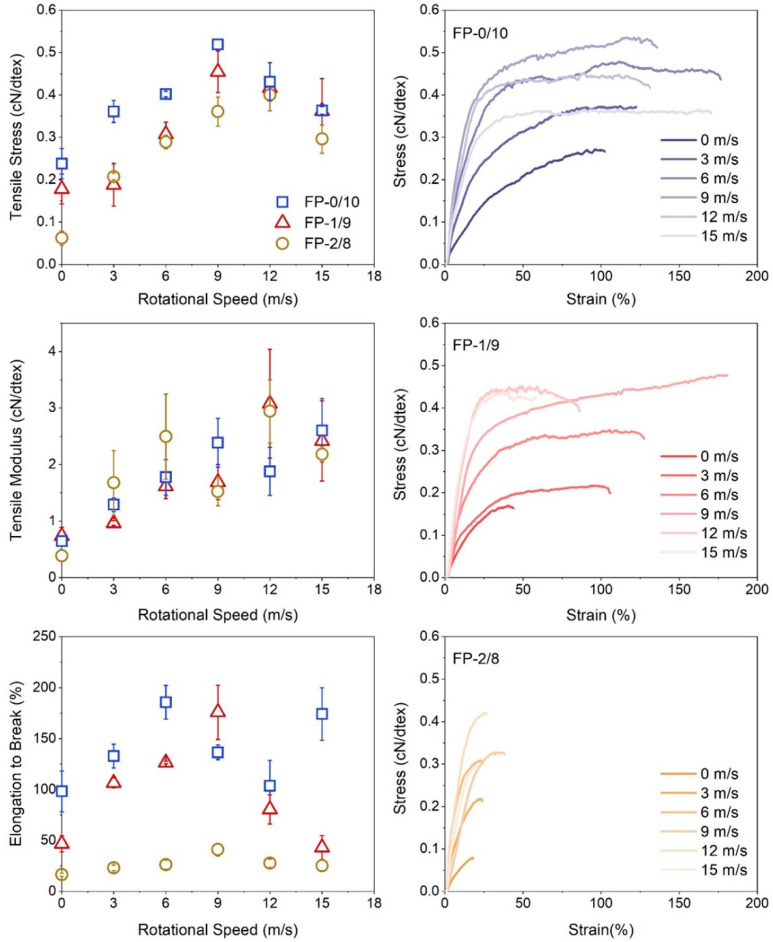
Tensile properties of the fabrics of FP-0/10, FP-1/9, and FP-2/10.

Generally, for monofilament, increasing the drawing speed results in the increase of tensile stress and the decrease of tensile modules and elongation to break, however, the tensile properties for the nonwoven fabrics could be more complex, which are related to the orientation intensity, the fiber morphology, and the interactions of fibers. Figure 3 shows the tensile properties of the above-mentioned oriented nanofiber fabrics. Broadly speaking, with increasing the rotational speed, the tensile stress and elongation to break increase first and then decrease and the turning point is close to the rotational speed of 9 m/s, while the tensile modulus shows linear increasing. It is known that the tensile strength of nonwoven fabric increases with the enhancement of fiber orientation intensity. Remember that the maximum orientation intensity occurred at the rotational speed of 12 m/s. The differences of the peaks suggest that the tensile strength of the composite nanofiber starts decreasing from the rotational speed of 9 m/s or from the rotational speed lower than 9 m/s, which further proves that the disturbed electrostatic field at high rotational speed is not beneficial for improving the mechanical properties of the nanofibers. In addition, mixing in 10 wt% of FNFs does not change the mechanical properties of PVA fabric too much. Even mixing up to 20 wt% of FNFs, the tensile strength and tensile modulus keep at ideal values, although its elongation to break dropped down to 20% caused by the beads-on-string fiber structure.

### 3.3. FTIR of the FNFs/PVA Nanofibers

**Figure 4:**
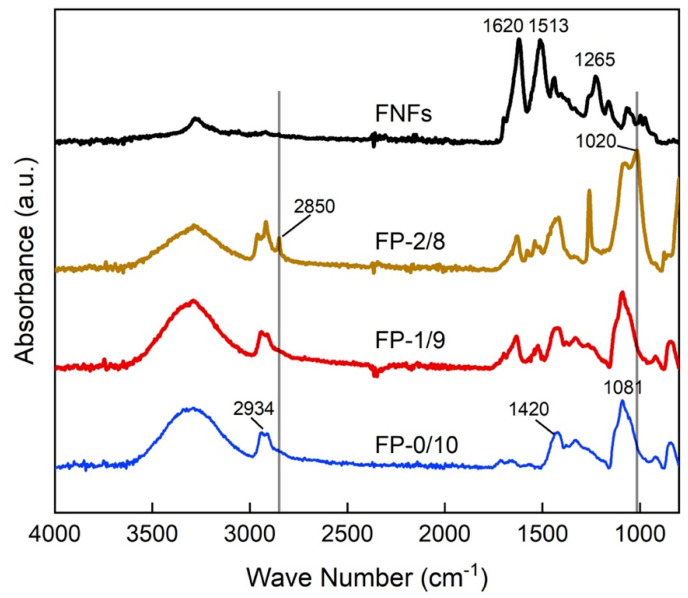
FT-IR spectra of the PVA, FNFs, and FNFs/PVA composite nanofibers.

FTIR spectra of PVA, FNFs, and their composites are shown in Figure 4. For PVA, a large peak ranging from 3550 to 3000 cm^−1^ and duplet absorption peak at 2934 cm^−1^ of PVA were attributed to O-H stretching and C-H alkyl stretching vibration, respectively [21]; the peaks at 1420 and 1081 cm^−1^ of PVA can be attributed to CHOH bending and C-O out-of-plane bending, respectively [22,23]. For FNFs, the absorption peak ranging from 3400 to 3100 cm^−1^ was caused by N-H stretching amide bonds; 1620 cm^−1^ was for C=O stretching vibrations in amide I (β-sheet structure); 1513 cm^−1^ was for N-H and C-N stretching vibrations in amide II (β-sheet structure); and 1265 cm^−1^ was for C-N and N-H stretching vibrations in amide III [23,24,25]. The FTIR spectra of the FNFs/PVA composites have the characteristic absorption peaks of both polymers. The FNFs derived peaks for amide I and II get stronger with the addition of the FNFs.

For the high FNFs contained fibers (FP-2/8), a new peak at 1020 cm^−1^ for C-O stretching vibration was significantly strengthened, and another new peak at 2850 cm^−1^ attributed to C-H stretching vibration from alkyl groups appeared in the vicinity of the duplet absorption peak at 2934 cm^−1^. These results suggest that the intermolecular hydrogen bonds between the hydroxyl group of PVA and the hydroxyl group of the amide group side chain, such as tyrosine and serine, exposed on the surface of FNFs occurred in FP-2/8.

### 3.4. Thermal Behavior of the FNFs/PVA Nanofibers

**Figure 5:**
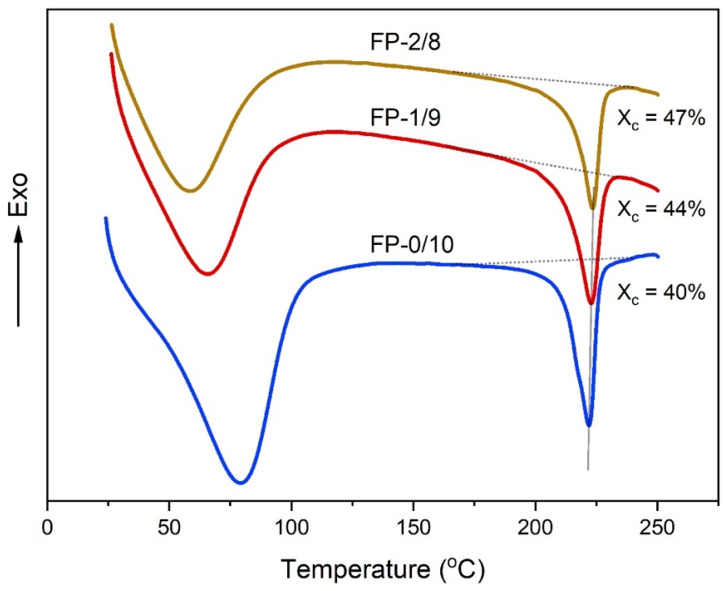
DSC curves of PVA and FNFs/PVA composite nanofibers.

Figure 5 shows the DSC curves of the composite fibers. Since FNFs belong to thermosetting polymer, the DSC curve will not show the exothermic or endothermic peak. The crystallinity of the PVA, calculated from the melting peak area, increased with increasing the content of FNFs. It was reported that the crystallinity of PVA was increased when cellulose nanofibers or carbon nanotubes were mixed [26,27]. Accordingly, the FNFs also can act as a nucleating agent that promotes the crystallinity of PVA. Moreover, the melting temperature (*T*_m_) of FP-0/10, FP-1/9, and FP-2/8 were detected at 222.1 °C, 222.9 °C, and 223.5 °C, respectively, that is, the *T*_m_ of PVA was slightly increased with increasing FNFs contents. However, previous studies suggested that the *T*_m_ of PVA was decreased with adding cellulose nanofibers or whiskers [28,29]. This is ascribed to the strong hydrogen bonding with the PVA and surface of cellulose which inhibit the capability of the PVA chains to increase the crystal size of PVA [29,30]. While some few hydroxyl groups on the surface of FNFs should not significantly restrict the alignment of the PVA chains. These results suggest that the crystallinity of PVA in electrospun nanofibers could be increased with the adding of FNFs.

**Figure 6:**
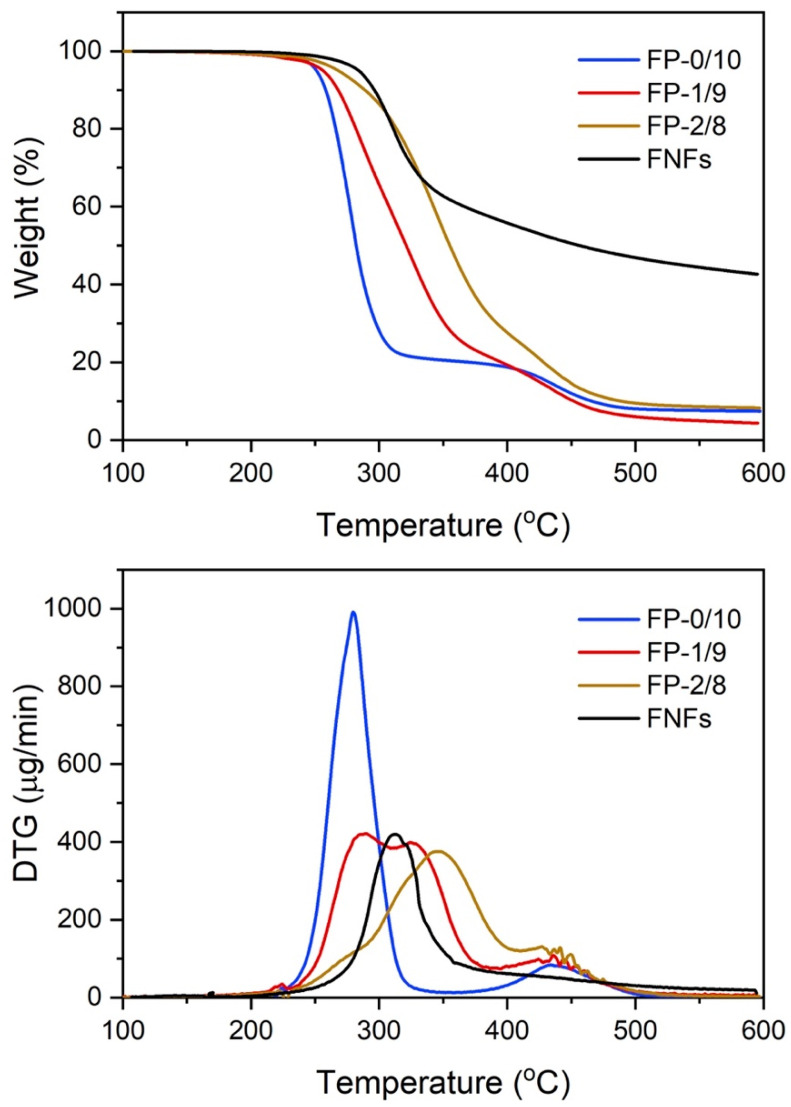
TG and DTG curves for PVA, FNFs, and FNF/PVA composite nanofibers.

The TG and DTG curves of PVA, FNFs, and their nanofiber composites are shown in Figure 6. The weight losses for PVA occurred at 210 °C and 400 °C, caused by the decomposition of the side chain and the main chains of PVA [31]. The pyrolysis temperature of the composite nanofibers was increased with the addition of FNFs. In the plot of DTG, FP-1/9 has two peaks at 240 °C and 350 °C due to the decomposition of PVA. However, these two peaks merged together when the FNFs increased to 20 wt% and shifted to a temperature even higher than that of FNFs. It is considered to be the fibrillar networks of FNFs in PVA matrix and hydrogen bonding between PVA and FNFs without any phase separation. Previous studies have reported that hydrogen bonding interactions plays an important role in improving polymer composites by promoting the miscibility of polymer blends [32]. Additionally, the increased crystallinity of PVA with the addition of FNFs made the molecular chains of PVA more rigid and pushed the DTG peak to a higher temperature.

## 4. Conclusions

Generally, the β-form crystal of fibroin will shift to amorphous after dissolving in good solvents, greatly reducing the mechanical properties of the regenerative fibroin, while through a simple grinding, the β-form crystal can be kept. In this study, a phase graph relate to the spinnability of FNFs/PVA was established. The mechanical properties of the composite nanofibers increased with increased fiber orientation while beads-on-string fibers occurred at high collector speed. When PVA was mixed with 20 wt% FNFs, intermolecular hydrogen bonds generated between these two polymers will greatly enhance the thermal properties of the composite nanofibers. The proposed strategy will be a typical method to regenerate the fibroin in a physical way. Comparing to be a film, the nanofiber FNFs/PVA composite nanofiber membrane with numerous pores will be a perfect scaffold to facilitate cells’ attachment, growth, and proliferation. These findings open a perspective to fabricate FNFs-based fibers that will facilitate the usage of silk in cutting-edge fields and extend the potential applications of the FNFs as well.

## Data Availability

The data presented in this study are available on request from the corresponding authors.

## Supplementary Materials

The following are available online at https://www.mdpi.com/article/10.3390/polym14010043/s1. Figure S1: WAXD of FNFs film, PVA, and FNFs/PVA nanofibers. Figure S2: The binary images of Figure 2. Figure S3: Plot of fiber orientation intensity versus rotational speed.
