# Supplementary material for: Reconstruction of Fibroin Nanofibers (FNFs) via Electrospinning: Fabrication of Poly(vinyl alcohol)/FNFs Composite Nanofibers from Aqueous Solution
Source: Polymers (Basel). 2021 Dec 23;14(1):43. doi: 10.3390/polym14010043 (PMC8747545; doi:10.3390/polym14010043)
Supplement: Supplementary file 1 [file polymers-14-00043-s001.zip › polymers-1497204-supplementary.pdf]

Supporting Information

Reconstruction of fibroin nanofibers (FNFs) via electrospinning: fabrication of poly(vinyl alcohol) / FNFs composite nanofibers from aqueous solution

Shohei FUJITA[1], Huaizhong XU*[1], Yubing Dong[2], Yoko OKAHISA*[1]

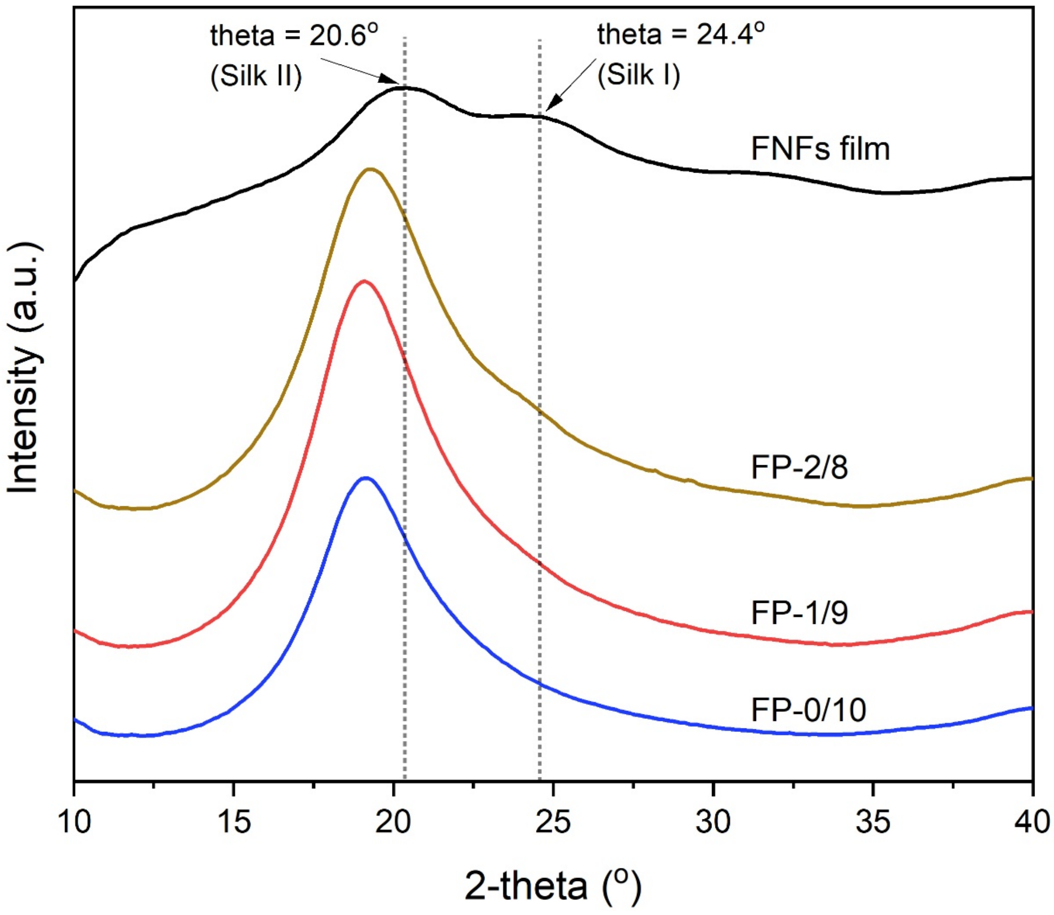

Figure S1. WAXD of FNFs film, PVA and FNFs/PVA nanofibers.

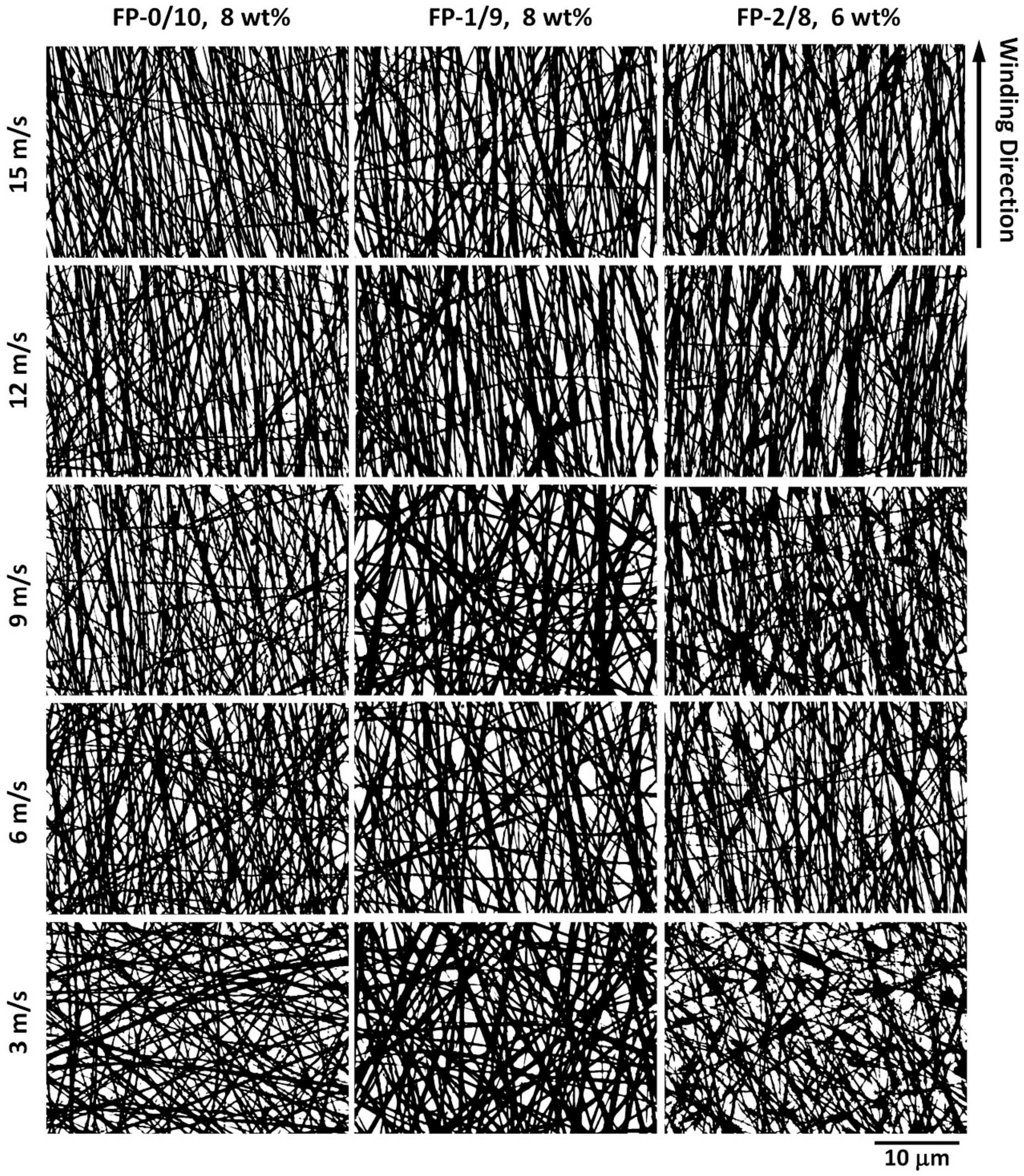

Figure S2. The binary images of figure 2.

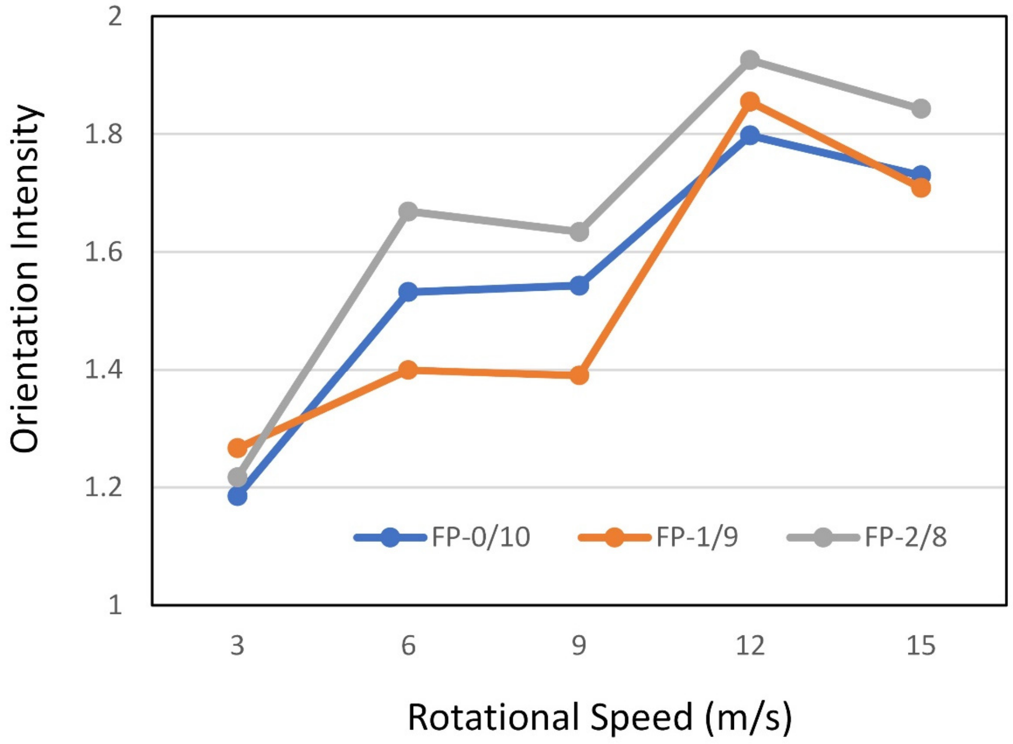

Figure S3. Plot of fiber orientation intensity versus rotational speed.
